# Nanocomposites of Au/Disentangled UHMWPE: A Combined Optical and Structural Study

**DOI:** 10.3390/molecules25143225

**Published:** 2020-07-15

**Authors:** Stavros X. Drakopoulos, Oreste Tarallo, Linlin Guan, Ignacio Martin-Fabiani, Sara Ronca

**Affiliations:** 1 Department of Materials, Loughborough University, Leicestershire LE11 3TU, UK; Linlin.Guan@manchester.ac.uk (L.G.); I.Martin-Fabiani@lboro.ac.uk (I.M.-F.); S.Ronca@lboro.ac.uk (S.R.); 2 Department of Chemical Sciences, University of Naples Federico II, 80126 Napoli, Italy; Oreste.Tarallo@unina.it; 3 School of Materials, University of Manchester, MSS Tower, Manchester M13 9PL, UK

**Keywords:** polymer nanocomposites, ultra-high molecular weight polyethylene, structural analysis, X-ray scattering, optical absorption, Au nanoparticles, orientation

## Abstract

The term disentangled refers to polymers with fewer entanglements in the amorphous regions, a metastable condition that can significantly affect the material’s properties and processing behavior. The lower entanglement density in ultra-high molecular weight polyethylene (dis-UHMWPE) facilitates the solid-state processability into uniaxially-oriented specimens reaching very high draw ratios and crystallinities. In this study, Au/dis-UHMWPE nanocomposites were formulated and processed at variable draw ratios. Polarized light microscopy suggests gold nanoparticles are oriented in arrays following the drawing of polymer chains. The structural features, upon orientation, are studied by means of Raman spectroscopy, wide- and small-angle X-ray scattering, and near-infrared spectrophotometry. Crystallinity is found to increase by 15%, as calculated by wide-angle X-ray scattering. The change in optical absorbance in the visible spectrum indicates that, with orientation, the average size of gold aggregates increases, supported quantitatively by small-angle X-ray scattering. Since the gold nanoparticles are expected to be found within amorphous chain segments, the aforementioned findings are attributed to the increase of crystallinity and thus the decrease of available (amorphous) space.

## 1. Introduction

In recent years, interest has been increasing in polymers characterized by a lower entanglement density, which can greatly affect the properties and processability of the material [1]. Advancements in chemical synthesis allowed the evolution of disentangled ultra-high molecular weight polyolefins like polyethylene and polypropylene, which possess enhanced thermal and mechanical properties [2,3,4]. A better understanding of the structure of these disentangled polymers could lead to breakthroughs in various high-end applications [5,6,7]. 

Ultra-high molecular weight polyethylene (UHMWPE) is a linear macromolecule produced by the polymerization of ethylene gas and is characterized by an average molecular weight in the order of 10^6^ g/mol or higher [8,9]. UHMWPE can be chemically synthesized by means of either a heterogeneous Ziegler-Natta catalyst [10] or homogeneous catalysts, such as metallocenes and post-metallocenes [11,12]. Homogeneous catalysts under controlled reaction conditions can produce macromolecules with a lower entanglement density (dis-UHMWPE) [5]. These polymers can be processed in the solid state, whereas their fully entangled versions can only be processed in a melted state [5,9,12]. Dis-UHMWPE is known for its metastable character, as the evolution of entanglements plays a paramount role in the observed properties and applications [13,14,15]. 

The effect of uniaxial orientation has on the physical properties and characteristics of polyethylene has been under investigation for the past 40 years [16,17,18]. Highly anisotropic stretched dis-UHMWPE samples can be solid-state-produced via compression molding, rolling, and tensile stretching [19,20,21]. Uniaxial orientation was found to significantly increase the tensile modulus and strength [22,23,24], and more recently, the thermal conductivity of UHMWPE samples, achieving metal-like thermal conductivities in the stretched direction [25,26]. A considerable increase in the thermal conductivity was observed even at relatively low draw ratios, where ballistic phonons contribute to the increased thermal conductivity over distances as far as 200 nm, according to transient grating spectroscopy [27]. In the presence of suitable fillers (metallic nanoparticles or conjugated molecules), orientation brings anisotropy to the optical properties as well. This is due to the alignment of the fillers and polymer chains, which result in anisotropic optical absorption [28,29,30]. The anisotropic optical absorption induced by uniaxial plastic deformation of UHMWPE modified with nanofillers can be employed in more durable polarizers. In this application, the hydrophobic character of polyethylene can play an additional beneficial role, ascribed to its durability in humid environments [31].

In the present work, we investigated the optical properties and structural changes in Au nanoparticles/dis-UHMWPE composites induced by uniaxial plastic deformation at low, medium, and high draw ratios. The morphology was observed by scanning electron microscopy (SEM) and the optical properties were studied by means of polarized light microscopy and visible (VIS) spectrophotometry. The structural features were realized by means of Raman, small- and wide-angle X-ray scattering (SAXS and WAXS, respectively), and near infrared (NIR) spectroscopies, and the results were discussed considering the role of crystallinity on the distribution and orientation of gold nanoparticles. 

## 2. Results and Discussion 

### 2.1. Morphology and Optical Properties

**Figure 1:**
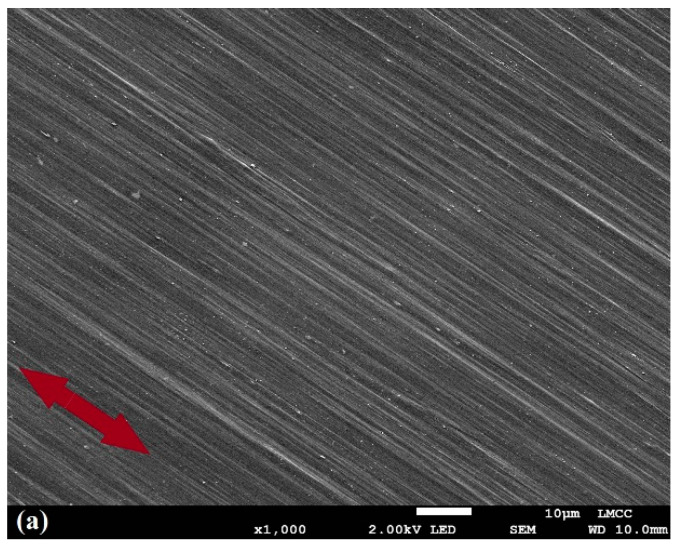

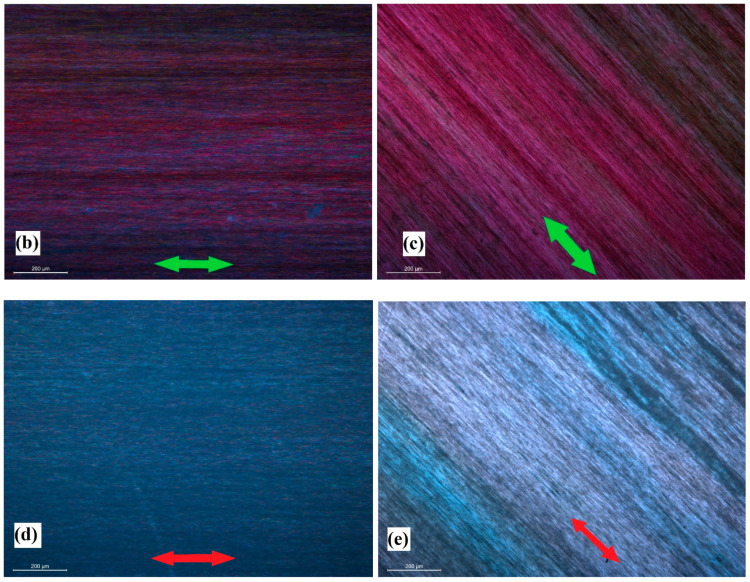
(**a**) SEM micrograph at 1000 magnification and (**b**–**e**) polarized light optical micrographs at two different angles for the medium draw ratio sample (DR100) with (**b**,**c**) the nanocomposites and (**d**, **e**) the plain dis-UHMWPE samples. The arrows show the orientation direction. (**b**,**d**) The polarized light is diagonal (45°) and (**c**,**e**) the polarized light is perpendicular (90°) to orientation direction.

The morphology of a medium draw ratio (DR100) sample was observed via SEM, which showed that the polymer phase was uniformly oriented along the stretching direction (Figure 1a). Because dis-UHMWPE is a strongly anisotropic material due to its orientation, crystalline polydispersion, and crystallinity variation, we expected that the gold nanoparticles, which reside only in the amorphous section of the polymer, would not be homogenously distributed on the nanoscale.

The dichroic optical properties of the sample were characterized by means of polarized light optical microscopy. Optical micrographs at two different polarizer angles are presented in Figure 1b,c. The micrographs exhibit very bright colors and, depending on the polarization direction of the incident light with respect to the stretching direction of the sample, appear blue or red. In particular, when the polarized light is diagonal (θ = 45°, Figure 1b) to the orientation, the image is a combination of blue and red shades, whereas when the polarized light is perpendicular (θ = 90°, Figure 1c) to the orientation, the image is just red. As pointed out by Dirix et al., in the case of analogous nanocomposites with high-density polyethylene modified with silver [32] and gold [33] nanoparticles, the polarization-dependent colors originate from a needle-like ordering of the spherical metal nanoparticles in the oriented polymer matrix. This effect was considerably enhanced by the presence of the gold nanoparticles, which can be highlighted by comparing Figure 1b,c with plain dis-UHMWPE samples at the same draw ratio and polarizer angles, presented in Figure 1d,e, respectively. In Figure 1e, a very weak discoloration can be detected and is attributed to the photoelastic effect.

**Figure 2:**
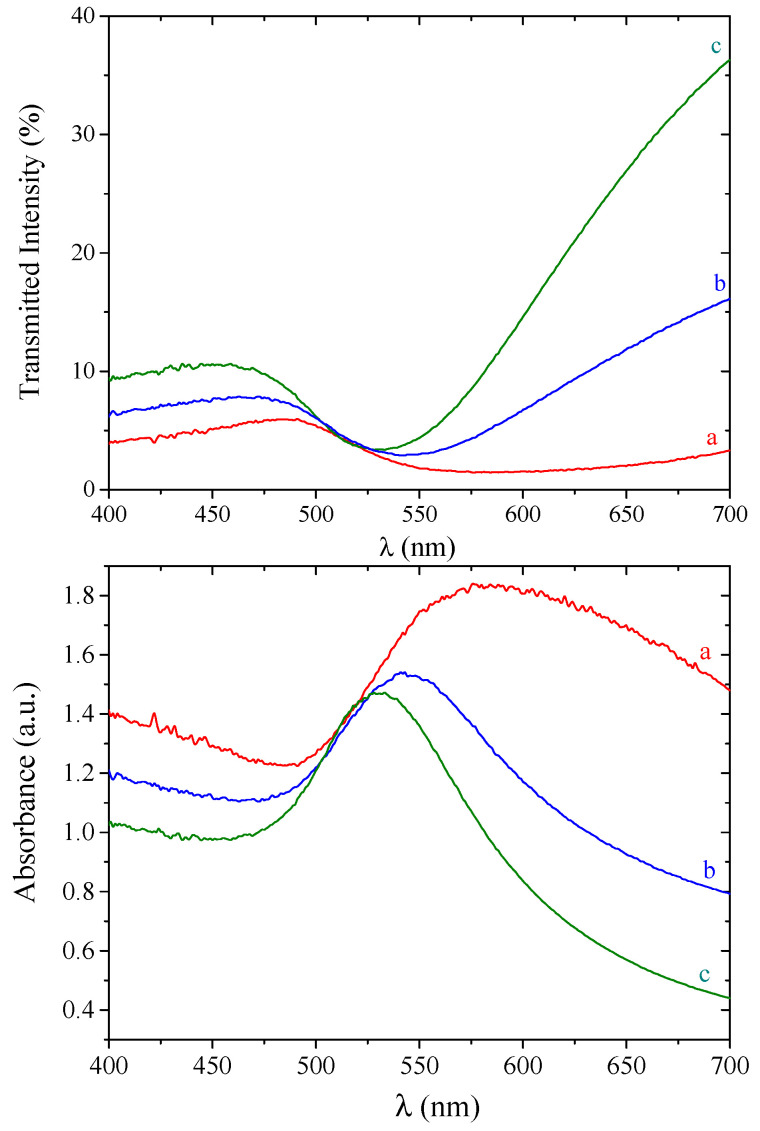
Transmittance/absorbance spectra of DR100 UHMWPE/gold nanocomposite in plane polarized light. The angle between the polarization direction of the light and the drawing direction of the film (θ) is (a) 0° (red curve), (b) 45° (blue curve), and (c) 90° (green curve).

These data are in agreement with the VIS transmission spectra of the DR100 oriented nanocomposite in polarized light, shown in Figure 2. For 400 ≤ λ ≤ 700 nm, the nanocomposite exhibited an absorption spectrum that is strongly dependent on wavelength, the angle between the polarization direction of the incident light, and the drawing direction of the sample. Despite a limited transmittance, probably due to its thickness (~50 μm) and its elevated draw ratio, which is likely to cause micro-voids, the sample showed a neat dichroic behavior. When the vibrating plane of polarized light was parallel to the drawing direction (θ = 0°), the absorption maximum was observed at 580 nm. However, in the case θ = 90°, i.e., when the vibrating plane of polarized light was perpendicular to the drawing direction, light was absorbed at lower wavelengths, with an absorption maximum of 398 nm. An isosbestic point was observed in the absorption spectra at a wavelength of 425 nm. 

This dichroic effect was not observed in the undrawn nanocomposites, as the transmittance of an oriented UHMWPE image of the same thickness but without metallic nanoparticle was ~40% and nearly constant over the entire VIS wavelength.

**Figure 3:**
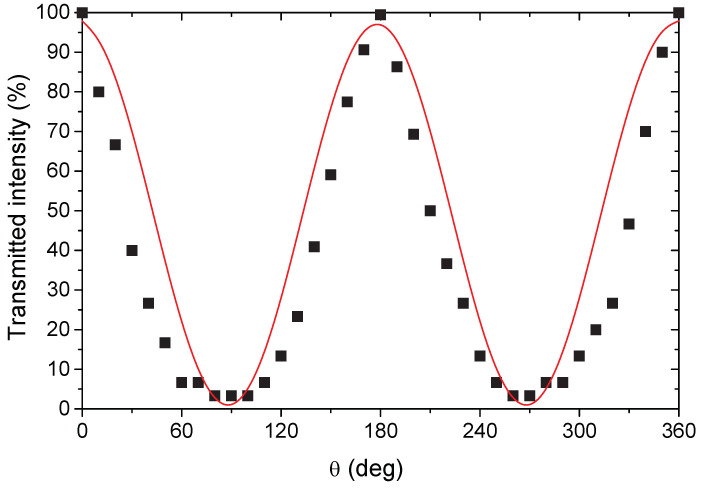
Transmitted intensity (black squares) in linearly polarized light for DR100 nanocomposite as a function of the angle θ between the polarization direction of the light and the drawing direction of the film. The red curve is a fit of experimental points according to Malus’ law.

Figure 3 shows the variation of the transmitted intensity for the same sample vs. θ. It is apparent that the collected data are in fairly good agreement with Malus’ law, and that the sample showed a good polarization capability (*p* ≈ 0.9).

**Figure 4:**
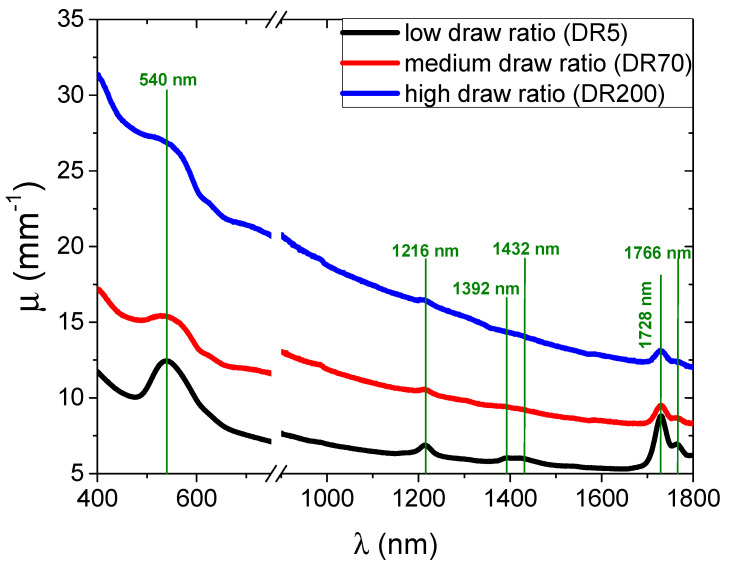
Linear absorption coefficient of the UHMWPE/gold nanocomposites as a function of wavelength with green lines showing relevant peaks.

The VIS/NIR absorption spectra of composites characterized by different draw ratios are presented in Figure 4. To normalize the results by sample thickness, the linear absorption coefficient, *μ*, is employed as presented in Equation (1) [34]:

(1) $$\mu =\frac{-ln\left(T\right)}{l},$$

where *T* is the fractional intensity of light transmission and *l* the thickness of the sample. At a wavelength range between 1000 and 1800 nm (NIR), the absorption at a low draw ratio exhibits five main peaks, all attributed to plain polyethylene [35,36]. It is evident that with tensile stretching, the absorption coefficient values increase in addition to a change in the peak shape. Orientation decreased the 1392 and 1432 nm (CH_2_ stretching) peaks to the point that they were no longer visible, ascribed to density enhancement resulting from the crystallinity increase [37]. The molecular mechanisms behind the rest of the peaks can be found elsewhere [37,38,39]. In the visible part of the spectrum, only one peak at 540 nm was visible and due to the transverse plasmon resonance of the gold nanoparticles that were located in the amorphous regions of the polymer [35]. The plasmon peak shifted toward longer wavelengths and became broader with increasing orientation, indicating an increase in the average size and polydispersity of the nanoparticle aggregates. A further confirmation of the formation of needle-like aggregates of nanoparticles was the appearance in the spectra of the high draw ratio samples of an additional resonance at λ that was red-shifted with respect to the maximum transverse plasmon resonance of gold nanoparticles (i.e., ~630 nm), due to the new longitudinal resonances available due to the formation of the oriented metallic needles dispersed in the UHMWPE matrix [29].

### 2.2. Raman Shift

**Figure 5:**
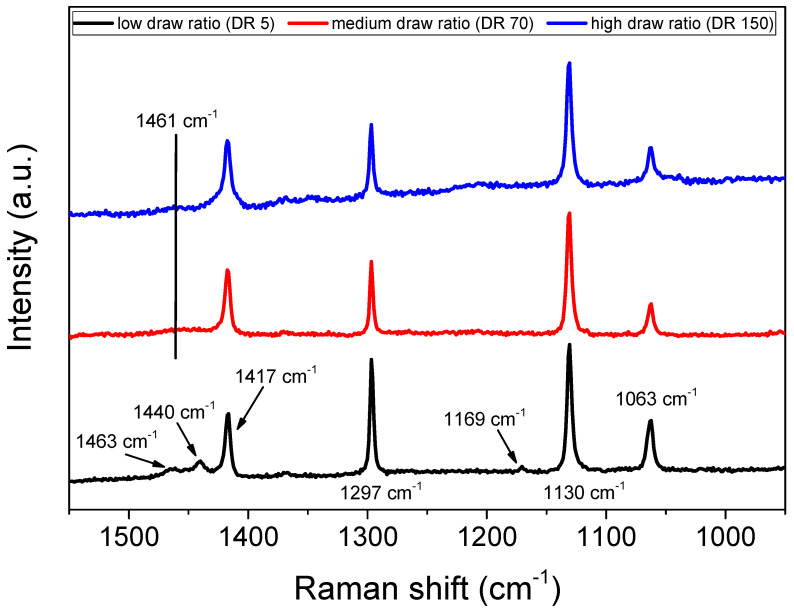
Normalized Raman spectra as a function of wavenumber varying draw ratios for UHMWPE/gold composites at different draw ratios. Each curve was normalized with the intensity of the 1130 cm^−1^ peak.

Uniaxial orientation induced structural changes in the molecular vibrations for draw ratios up to 10, as previously shown with Raman spectroscopy [40]. In Figure 5, the Raman shift of the highly oriented samples is presented. The skeletal C–C stretching vibrations region was found between 1160 and 1000 cm^−1^, where two narrow peaks were observed at 1130 and 1063 cm^−1^, representing the in-phase and out-of-phase vibrational modes, respectively [41]. Although mostly related to the crystalline phase, there is a contribution from the amorphous phase in these two modes [40]. The intensity of these peaks was not affected at all by tensile stretching compared to the low orientation sample. The small peak at 1169 cm^−1^, related to the CH_2_ rocking mode and exhibiting contributions from both the crystalline and the amorphous phases, appeared to vanish with increased drawing. The twisting vibration of CH_2_ coming from the crystalline phase was observed at 1297 cm^−1^ and slightly decreased with stretching. 

The CH_2_ bending vibrational mode was split into two contributions, at 1440 and 1417 cm^−1^, which is indicative of an orthorhombic crystal structure [42]. Rolling was proven to significantly affect the intensity of these three peaks; when rolled, the 1417 cm^−1^ crystalline peak increased, whereas the 1463 and 1440 cm^−1^ amorphous peaks decreased [43]. For the medium and highly oriented samples, we observed that the two amorphous peaks at 1463 and 1440 cm^−1^ vanished completely and a very broad amorphous halo at 1461 cm^−1^ replaced them, which indicated the decrease in amorphous content. This change also indicated a crystallinity increase, which was confirmed quantitatively by WAXS analysis, as noted below.

### 2.3. Wide-Angle X-ray Scattering

**Figure 6:**
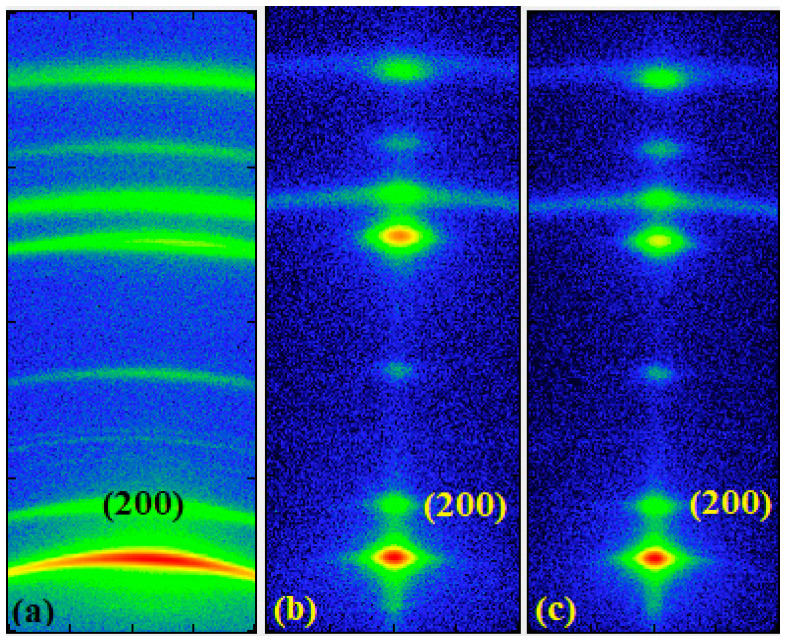
2D WAXS patterns of oriented UHMWPE/gold nanocomposites at (**a**) low (DR5) draw ratio, (**b**) medium (DR70) draw ratio, and (**c**) high (DR150) draw ratio. The horizontal axis corresponds to the drawing direction.

To understand the effect of tensile stretching upon the crystalline structure of UHMWPE, wide-angle X-ray scattering was carried out. In Figure 6, the 2D WAXS patterns are presented, where it can be observed that the anisotropy of the patterns increased with increasing orientation. The low draw ratio sample already shows signs of orientation because its WAXS pattern is characterized by scattering reflections, which show a non-uniform intensity that is slightly concentrated in the meridian of the 2D pattern (Figure 6a). As the draw ratio increased, reflections concentrated further into spots, indicating a high degree of orientation for high draw ratios. 

**Figure 7:**
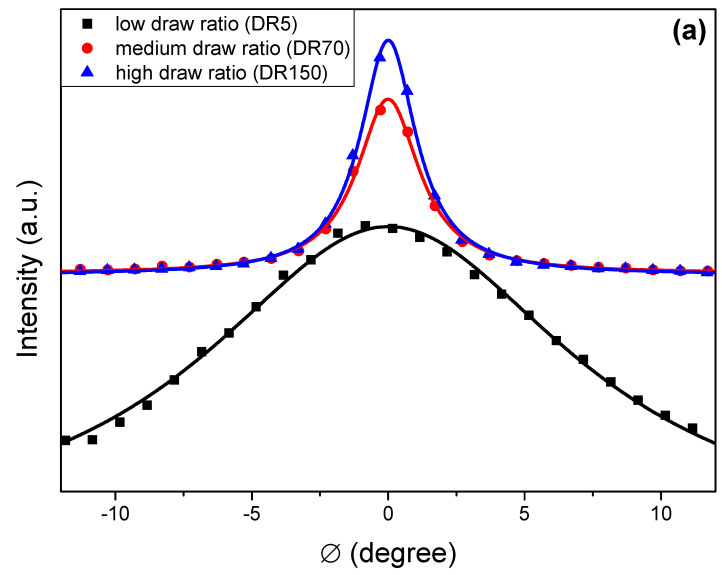

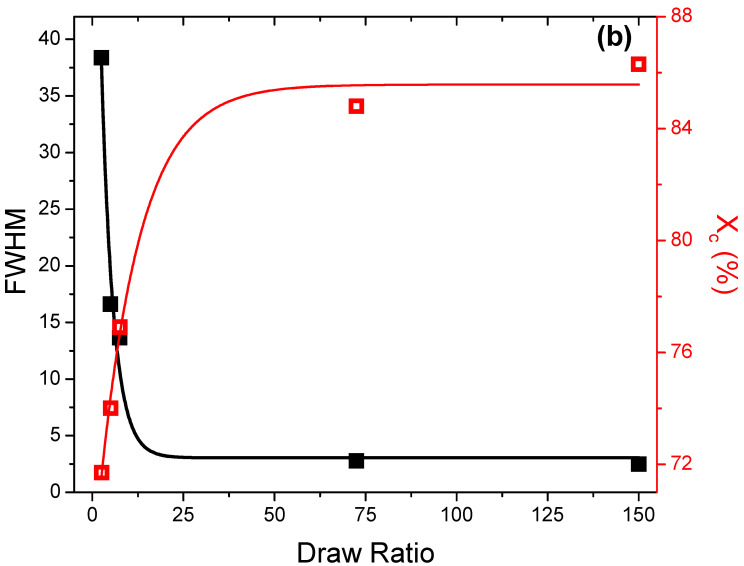
(**a**) Intensity as a function of azimuthal angle Ø for the (200) diffraction peak for the DR5, DR70, and DR150 samples. (**b**) The full-width at half maximum (FWHM) of the (200) reflection as obtained from the azimuthal profiles and the sample crystallinity is presented, including the DR2.5 and DR7.5 samples as a function of draw ratio. The lines (a) are Lorentzian fits and (b) guides for the eye only.

The degree of crystallinity of the composites was calculated by dividing the area below the crystalline peak, obtained by Lorentzian fits of the integrated 2D patterns, by the total area of crystalline and amorphous halos. A radial integration for a narrow q-range was performed on the (200) reflection to obtain the scattered intensity as a function of azimuthal angle Ø, as shown in Figure 7a. Then, these profiles were fitted using Lorentzian functions to obtain their full-width at half maximum (FWHM) as shown in Figure 7b. The (200) reflection became more intense and narrower as orientation increased, with the FWHM reaching a plateau for high draw ratios. Crystallinity (Figure 7b) increased from 75% at low draw ratios to 87% at high draw ratios. The aforementioned values are in agreement with those reported by solid-state NMR experiments performed on similar dis-UHMWPE samples, where an increase in crystallinity, from 75% to 90%, was observed for samples with draw ratios between 0 and 140 [21].

### 2.4. Small-Angle X-ray Scattering

**Figure 8:**
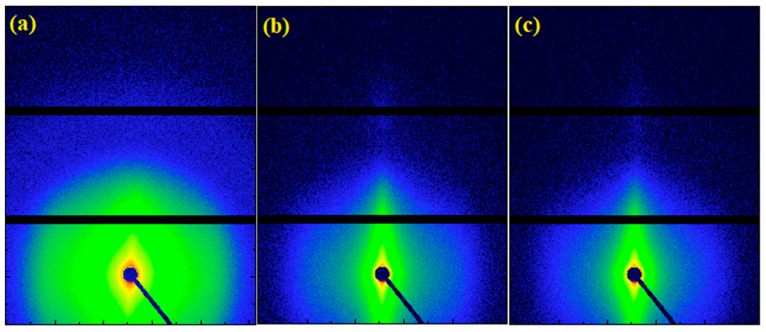

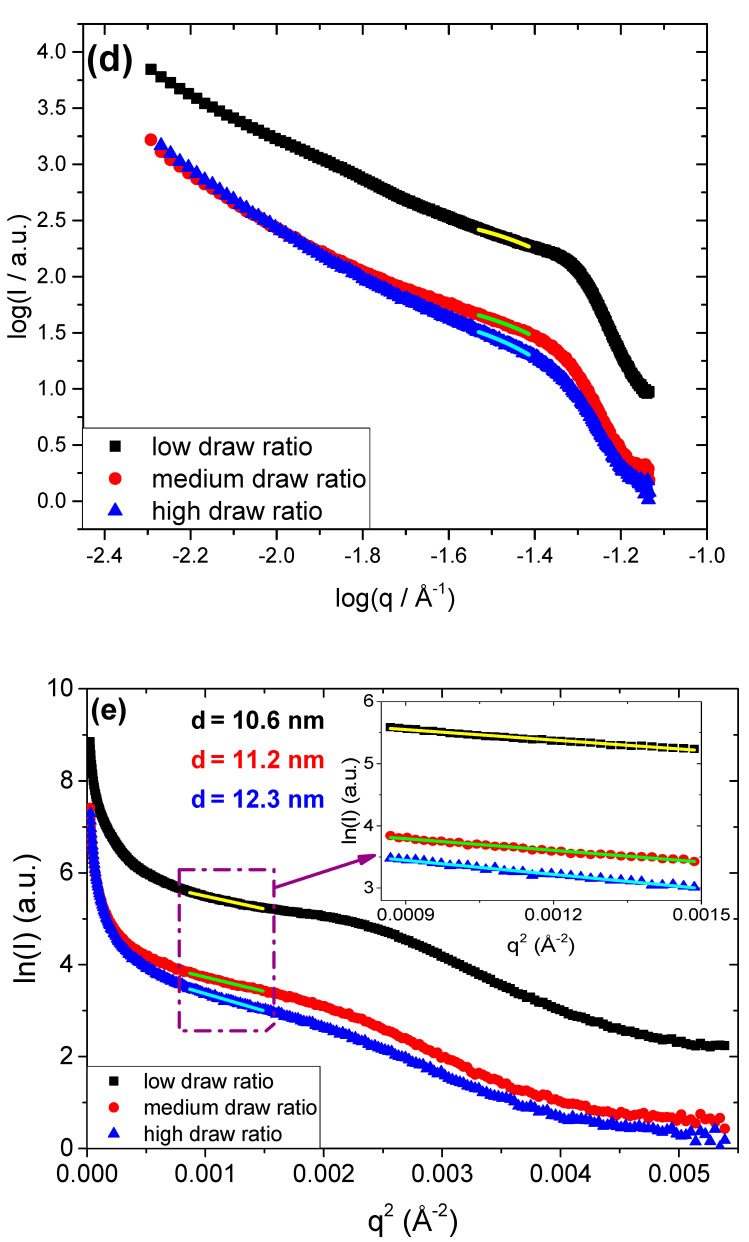
2D SAXS patterns of UHMWPE/gold nanocomposites at (**a**) low draw ratio (DR5), (**b**) medium draw ratio (DR70), and (**c**) high draw ratio (DR150). After integration over all azimuth angles, we obtained the 1D profiles in (**d**) log-log representation and (**e**) Guinier plot of the SAXS patterns, fitted with the Guinier law. The diameters of the gold nanoparticles as obtained by the Guinier law for spherical nanoparticles are presented within the graph.

The 2D SAXS patterns are presented in Figure 8a–c, which follow a similar trend as the WAXS patterns; anisotropy is more prominent as the draw ratio increases. Orientation brought an increase in intensity close to the meridian, as analyzed extensively previously [44]. In Figure 8d, the gold aggregates have a very strong scattering contribution in the low q-range due to their significantly higher electron density with respect to that of polyethylene. Hence, the presence of gold nanoparticles masked the lamellae long spacing of polyethylene, which was not visible in any of the samples under study. To calculate the average diameter of the gold nanoparticles, the Guinier law for spherical particles of diameter *d* was used [45]:

(2) $$I\left(q\right)e^{\frac{-q^{2}R_{g}^{2}}{3}}$$

(3) $$d=2\sqrt{\frac{5}{3}}R_{g}$$

where *R_g_* is the radius of gyration of the nanoparticle. From this analysis, the average size of the nanoparticles is presented in Figure 8e, which was found to be 10.6, 11.2, and 12.3 nm for the low, medium, and high draw ratios, respectively. This suggested an increase in the nanoparticle aggregate size. The Guinier law was employed here to describe the nanoparticles as an approximation to estimate their average diameter, although the shape of the nanoparticles was not expected to be perfectly spherical. Because nanoparticles can only lie in the amorphous regions of the polyethylene matrix, the increase of the aggregate’s diameter was attributed to a reduction of the amorphous phase fraction with orientation as crystallinity increased, resulting in less available space for the nanoparticles. If the crystallinity was not affected by orientation, we would expect the average size of the particles to decrease due to the breaking of aggregates; this was the case with very low draw ratios (≤DR 5), where the crystallinity only slightly increased.

## 3. Materials and Methods

### 3.1. Materials 

Dis-UHMWPE was synthesized in-house according to procedures described elsewhere [46]. The polymerization reaction was quenched with acidified methanol (methanol/37% *w*/*w* HCl 95/5 *v*/*v*) to completely avoid the formation of Al_2_O_3_ catalytic ashes from the methyaluminoxane (MAO) co-catalyst used. The average molecular weight (*M*_w_) was determined by rheological measurements to be *M*_w_ = 5.6 × 10^6^ g/mol [47]. Dodecanethiol-functionalized gold nanoparticles dissolved in toluene (2% *w*/*v*) were purchased from Sigma-Aldrich (Darmstadt, Germany) and used as received. The distribution of nanoparticle diameters, as given by the supplier, was 2–5 nm.

### 3.2. Specimen Manufacturing

Dis-UHMWPE in powder form was suspended in acetone and, after 1 h of magnetic stirring, the dodecanethiol-Au nanoparticles toluene solution was added under magnetic stirring. The stirring was maintained for a few hours and then the solvents evaporated under a fume hood overnight. Heating the nanocomposites at 50 °C under a vacuum for 6 h ensured the complete removal of residual solvents. The temperature was kept below the activation temperature for the formation of entanglements (58 °C) [14]. The dodecanethiol-Au nanoparticles concentration in dis-UHMWPE was kept constant at 1.0% *w*/*w*. Initially, specimens were prepared by means of compression molding, then uniaxial plastic deformation was induced by means of calendering, and when required, further orientation was achieved by means of tensile stretching. During all the processing steps, the temperature was kept below the melting point (~137–140 °C), at 125 °C. The machines’ temperature overshoot did not exceed 130 °C. The protocol for compression molding can be found elsewhere [14]. The low draw ratios were achieved by calendering to ratios up to 10 times; medium draw ratios were achieved by 50–100 repetitions; and high draw ratios (above 100) were realized by tensile stretching in a Hounsfield tensometer at 50 mm/min. The samples under study are summarized in Table 1.

### 3.3. Materials Characterization

#### 3.3.1. Scanning Electron Microscopy

The surface morphology of the oriented Au/dis-UHMWPE samples was inspected by a scanning electron microscope (JEOL 7800, Welwyn Garden City, UK), at 2 kV. Prior to the experiment, the samples were coated with a gold/palladium alloy. The typical dimensions of gold/palladium particles produced during sputtering were expected to be less than 10 nm in diameter.

#### 3.3.2. Polarized Light Microscopy

The change in optical properties with stretching direction (angle) was evaluated by means of polarized light microscopy, where the images were collected in transmission using a LEICA DFC480 (Hamburg, Germany).

The polarizing degree on oriented samples was studied by means of an in-house cross-polarized apparatus. The polarizer was a commercial polarizing filter(Hoya Polarising Linear Filter 58mm, Amsterdam, The Netherlands), whereas the examined samples were used as an analyzer. The degree of polarization *P* was determined by Equation (4):

(4) $$P=\frac{I_{max}-I_{min}}{I_{max}+I_{min}}$$

where $I_{max}$ and $I_{min}$ are the maximum and minimum intensity of the transmitted light, respectively, recorded upon varying (by 10 degree steps) the dihedral θ angle between the polarization plane of the light (λ = 635 nm) used to irradiate the sample and the stretching direction of the sample itself. Transmitted intensity was fitted according to Malus’ law, with a cosine squared function:

(5) $$y=C+Acos^{2}\left(x+B\right)$$

where y is the transmitted intensity, x represents the θ angle between the light’s initial polarization direction and the axis of the polarizer, A represents the amplitude of the square cosine function, B is the angular correction of the phase, and C is a background constant. 

#### 3.3.3. Vis/NIR Spectrophotometer

The optical absorption of the samples was investigated in the visible wavelength range of 400–750 nm and at the near infra-red wavelength of 950–1800 nm at room temperature. The equipment employed was a Cary 5000 UV-Vis-NIR spectrophotometer provided by Agilent Technologies (Santa Clara, CA, USA). Light was polarized by means of a commercial Polaroid filter (Hoya Polarising Linear Filter 58mm, Amsterdam, The Netherlands) mounted on a homemade support.

#### 3.3.4. Raman Spectroscopy 

The samples were analyzed in the wavenumber range of 1500–1000 cm^−1^ at room temperature employing a laser beam at 633 nm. The equipment employed was a LabRAM HR provided by Horiba Jobin-Yvon (Palaiseau, France).

#### 3.3.5. SAXS/WAXS Analysis

Simultaneous experiments of small- and wide-angle X-ray scattering were performed employing a Xenocs Xeuss 2.0 (Xenocs, Grenoble, France) equipped with a micro-focus Cu-Kα source collimated with scatterless slits. WAXS was measured on a Pilatus 100k (Baden-Daettwil, Switzerland) mounted at an angle of 36° to the beam direction at a distance of 162 mm. This gave a *q*-range for the detector of 1.3–3.3 Å^−1^. SAXS was measured using a Pilatus 300k detector (DECTRIS, Baden, Switzerland) with a pixel size of 0.172 × 0.172 mm. The distance between the detector and the sample was calibrated using silver behenate (AgC_22_H_43_O_2_), giving a value of 247 cm. The magnitude of the scattering vector (*q*) is given by *q* = 4πsin *θ*/*λ*, where 2*θ* is the angle between the incident and scattered X-rays and *λ* is the wavelength of the incident X-rays. The analysis was conducted by means of a Foxtrot light scattering software program.

## 4. Conclusions

In the present work, dis-UHMWPE samples reinforced with gold nanoparticles were prepared at low, medium, and high draw ratios. The effect of orientation upon the structural characteristics of the samples was investigated by means of Raman spectroscopy, wide- and small-angle X-ray scattering, and near-infrared spectrophotometry. The presence of the Au nanoparticles and orientation affected the optical properties as observed via visible spectrophotometry, with the nanoparticles being oriented in arrays, as seen by polarized light microscopy. Crystallinity increased (~15%) with orientation, as evaluated qualitatively by Raman spectroscopy and quantitatively by wide-angle X-ray scattering. The average Au nanoparticle aggregate size also increased, as calculated by means of small-angle X-ray scattering. In addition, the optical absorbance of the samples was evaluated in the visible and near infrared spectrum. The experimental data obtained by SAXS, WAXS, and optical absorbance in the visible spectrum indicated that the gold nanoparticle aggregates increase in size with orientation, an effect that we attributed to a reduction of the amorphous phase fraction where the nanoparticles lie. 

## Figures and Tables

**Table 1:** Uniaxial oriented draw ratios for the Au/dis-UHMWPE nanocomposites.

Name	Draw Ratio	Categories
DR2.5	2.5	Low
DR5	5.0	
DR7.5	7.5	
DR70	70.0	Medium
DR100	100.0	
DR150	150.0	High
DR200	200.0	
